# Modulation of Prostate Cancer Cell Behavior Through Acetylsalicylic Acid‐Treated Prostate Epithelial Cell‐Derived Conditioned Medium

**DOI:** 10.1002/cbf.70276

**Published:** 2026-08-05

**Authors:** Dilşad Özerkan, Selda Durukan, Mahsa Koucheki, Aysel Nesil

**Affiliations:** ^1^ Department of Genetic and Bioengineering, Faculty of Engineering and Architecture Kastamonu University Kastamonu Turkey

**Keywords:** acetylsalicylic acid, anti‐cancer, aspirin, chemopreventive, prostate cancer, secretome

## Abstract

Acetylsalicylic acid (ASA, aspirin), derived from salicylic acid in willow bark, is known for its analgesic, anti‐inflammatory, and antiplatelet properties. Beyond cardiovascular protection, increasing evidence suggests that ASA may reduce the risk of various cancers, including colorectal and prostate cancer. In this study, the effect of ASA‐treated prostate epithelial cells (RWPE‐1) on the proliferation and migration of prostate cancer cells was investigated. RWPE‐1 cells were exposed to different ASA concentrations, and non‐toxic concentrations were selected. Conditioned media (CM) from untreated and ASA‐treated RWPE‐1 cells were applied to PC‐3 prostate cancer cells. Cell viability was assessed using the MTT assay, while migration was evaluated using the wound‐healing assay, and colony formation was also assessed. ASA at 50 μM showed no cytotoxic effect on RWPE‐1 cells, while CM1 (ASA 50 μM) reduced PC‐3 cell viability to 60%. Moreover, CM1 (ASA 50 μM) and CM2 (ASA 25 μM) significantly inhibited PC‐3 cell migration. Comparing CM groups revealed that ASA exposure alters the secretome of prostate epithelial cells, thereby reducing cancer cell viability and motility. These findings suggest that ASA may serve as a promising chemopreventive and therapeutic agent for prostate cancer.

## Significant Statement

This study reveals that conditioned medium from aspirin‐treated prostate epithelial cells (RWPE‐1) significantly suppresses prostate cancer (PC‐3) cell viability, colony formation, and migration, without harming normal cells. Specifically, CM1 (ASA‐50 Μm) reduced PC‐3 viability to 60%, suggesting that it exerts indirect anticancer effects via epithelial cell–secreted factors. These findings highlight a novel mechanism underlying aspirin's chemopreventive potential against prostate cancer and emphasize the importance of the prostate epithelial microenvironment in modulating tumor progression.

## 1 Introduction

Prostate cancer is a disease of old age, with the highest incidence in men around 70 years of age, except for hereditary cases [1]. Based on data from the World Health Organization (WHO) GLOBOCAN, it ranks as the second most common cancer among men globally. It is also the second leading cause of cancer‐related fatalities, after lung cancer. Men have an approximately one in eight chance of being diagnosed with prostate cancer during their lifetime, and the likelihood of dying from the disease is roughly 1 in 37 [2]. Prostate cancer is quite rare in men under the age of 40, and cases in this age group account for only a tiny percentage of all prostate cancer cases [3].

The human prostate is a gland located just below the bladder and is composed of glandular units known as acini, supported by a fibromuscular stroma. The epithelial compartment of the prostate consists primarily of two main cell types: basal cells and luminal secretory cells. Basal cells form a discontinuous layer along the basement membrane, whereas luminal cells line the inner surface of the acini and are responsible for secretory functions. Prostate epithelial stem and progenitor cells give rise to transit‐amplifying (TA) cells, which can differentiate into both basal and luminal cell lineages, maintaining epithelial homeostasis. In prostate cancer and its early stages, this organized epithelial architecture becomes disrupted, leading to increased cellular proliferation, loss of basal cell layer integrity, and expansion of luminal‐like tumor cells. Prostate tumors are typically characterized by a predominance of luminal phenotype, basement membrane alterations, and a reactive stroma that supports tumor progression [4].

Aspirin (its active ingredient is acetylsalicylic acid [ASA]) is a non‐steroidal anti‐inflammatory drug [5]. Recently, especially with the acceleration of drug repurposing studies, the effects of aspirin, which is commonly used in daily life, on cancer have also been a matter of curiosity, and some studies have been conducted on this subject. For example, Several studies have proven that aspirin reduces the risk of colorectal cancer [6, 7]. It is also thought that aspirin may be protective against many types of cancer [8, 9, 10]. In a meta‐analysis, it was supported that the risk of developing gastrointestinal system cancers, including prostate cancer, was inversely associated with high or moderate aspirin use [11, 12]. According to individual patient data from randomized trials, aspirin has been shown to lower cancer incidence in both men and women [8].

At the cellular and molecular level, aspirin has been shown to modulate multiple signaling pathways involved in tumorigenesis. In cancer cells, ASA can inhibit prostaglandin‐mediated inflammatory signaling, suppress NF‐κB activation, alter platelet–tumor interactions, and influence pathways related to cell proliferation, apoptosis, and migration [13, 14]. However, most experimental studies investigating the anticancer properties of aspirin have focused on its direct effects on tumor cells. In contrast, considerably less attention has been given to how aspirin might influence non‐malignant cells within the tumor microenvironment.

The tumor microenvironment plays a crucial role in regulating cancer cell behavior through soluble factors, cytokines, growth factors, and extracellular vesicles secreted by surrounding cells. These secreted molecules collectively constitute the cellular secretome and mediate paracrine communication between epithelial, stromal, and tumor cells. Alterations in the secretome of non‐cancerous cells may therefore significantly influence tumor proliferation, survival, and migration. One plausible mechanism by which aspirin may indirectly affect cancer cells is by altering the secretory profile of normal epithelial cells. By inhibiting COX‐mediated prostaglandin synthesis and modulating inflammatory signaling pathways such as NF‐κB, aspirin may alter the release of cytokines, chemokines, and growth‐regulatory molecules from epithelial cells. Such changes could influence tumor cell behavior via paracrine epithelial signaling [15]. However, experimental studies investigating whether aspirin can modify the secretome of non‐cancerous prostate epithelial cells and thereby influence prostate cancer cells remain limited.

Therefore, the present study aimed to investigate whether aspirin pretreatment of normal prostate epithelial cells (RWPE‐1) alters their conditioned medium (CM) and whether these changes influence prostate cancer cell behavior. For this purpose, RWPE‐1 cells were treated with various non‐cytotoxic concentrations of ASA, and the CM obtained were subsequently applied to PC‐3 prostate cancer cells. The effects of ASA‐modified CM on cancer cell proliferation and migration were evaluated using MTT and wound healing assays. By focusing on the indirect effects of aspirin mediated through epithelial cell secretions, this study provides a microenvironment‐oriented experimental approach that differs from conventional models investigating direct drug–tumor interactions. Understanding whether aspirin can influence tumor behavior through epithelial‐derived paracrine signaling may contribute to a broader understanding of its potential chemopreventive and therapeutic roles in prostate cancer.

## 2 Methods

### 2.1 Preparation of ASA Compound

ASA stock and working solutions were freshly prepared before each experiment, and exposure times were limited to minimize hydrolysis to salicylate, which could confound the interpretation of ASA‐specific effects. The stock solution of ASA was prepared by dissolving 552 mg of ASA in 4 mL of dimethyl sulfoxide (DMSO), yielding an approximately 766 mM master stock. Working concentrations were freshly prepared by diluting the stock solution in culture medium. The final DMSO concentration in the highest treatment group (50 µM ASA) was approximately 65 µM, and was proportionally lower in the other treatment groups. Equivalent concentrations of DMSO were used in control groups to exclude solvent‐related effects. The prepared stock solution was stored in aliquots of 50, 25, 10, 5, and 2 μM at −20°C in the dark. The ASA concentrations used in this study (2–50 μM) were selected to represent pharmacologically relevant exposure levels. Pharmacokinetic studies have shown that low‐ concentration aspirin used for cardioprotection (75–100 mg/day) produces peak plasma concentrations of approximately 0.5–2.2 μg/mL, corresponding to roughly 3–12 μM ASA. In contrast, anti‐inflammatory therapeutic concentrations (4–8 g/day) produce substantially higher systemic salicylate levels in the range of 150–300 μg/mL. Therefore, the micromolar concentrations used in this study are consistent with physiologically achievable exposure levels associated with low‐concentration aspirin therapy [15, 16]. No sedimentation was observed after dilution. The ASA used in the study was supplied by Sigma (A5376‐100G), and the Dimethyl sulfoxide (DMSO) used as a solvent for cell biology was supplied by Neofroxx (1264‐ML100).

### 2.2 Cell Culture Analyzes

In this study, PC‐3 prostate cancer cells (ATCC, CRL‐1435) were cultured using Dulbecco's Modified Eagle's Medium (DMEM, THERMO‐41966029), whereas RWPE‐1, a prostate epithelial cell line, was cultured in RPMI‐1640 (THERMO‐21875034) medium. A suitable environment for cell culture was prepared by adding 10% Fetal Bovine Serum (FBS, PANBIOTECH P30‐3306) and 1% Penicillin‐Streptomycin (THERMO 15140122) solution to both media. The cells were grown under standard conditions in a 37°C incubator containing 5% CO_2_. To confirm the absence of mycoplasma contamination, routine Hoechst staining was performed on all cell lines as part of the regular quality control procedure.

Cell counting was performed by taking 10 μL of cells suspended after trypsinization. An equal volume of trypan blue was added to this cell suspension and mixed homogeneously. After the hemocytometer was cleaned and dried with 70% ethanol, 10 μL of the cell‐dye mixture was taken and carefully placed on one side of the slide. Under the microscope, the cells in the middle of the slide were counted. The number of cells obtained was averaged and multiplied by the dilution coefficient to calculate the total number of cells in 1 mL medium.

### 2.3 CM Harvest

CM is a milieu containing various biological components secreted by cells, such as cytokines, chemokines, and growth factors. This method offers a simple model of three‐dimensional microsystems that reflect cell interactions. In this study, CM obtained from RWPE‐1 prostate epithelial cells was applied to PC‐3 prostate cancer cells, and the effects of released factors on cancer cells were investigated. RWPE‐1 cells were seeded into 6‐well culture plates at a density of 5 × 10^4^ cells per well. Two experimental paths were followed for CM preparation:

1. *Control group:* One day after seeding, cells were switched to serum‐free medium without any pretreatment.
2. *ASA pretreatment groups:* One day after seeding, cells were treated with non‐cytotoxic concentrations of ASA (50, 25, 10, 5, 2 μM) for 24 h. Following pretreatment, cells were washed and cultured in serum‐free medium for an additional 24 h.

Serum starvation was performed uniformly across all experimental and control groups to ensure comparable baseline conditions. Serum contains numerous exogenous proteins and growth factors that could mask or influence effects observed in CM; thus, serum‐free conditions provide a standardized baseline for more accurate assessment of secretome‐mediated effects. Importantly, we confirmed that this 24 h serum deprivation did not adversely affect cell viability in our system, consistent with prior reports indicating that short‐term serum withdrawal reduces proliferation signals without inducing significant apoptosis or loss of viability, as confirmed experimentally or rigorously controlled [17].

After the 24‐h serum‐free incubation, the culture media from both groups were collected, transferred to 15 mL Falcon tubes, and centrifuged at 2000 rpm for 5 min to remove cellular debris. The clarified supernatant was carefully transferred to new tubes without disturbing the pellet, yielding CM samples from ASA‐pretreated and untreated RWPE‐1 cells. These CM samples were applied to PC‐3 cells at a final concentration of 50% (v/v) in complete culture medium. The CM groups were defined as follows (see Table 1).

**Table 1:** CM groups from RWPE‐1 cells pretreated with different ASA concentrations. Final CM was applied to PC‐3 cells at 50% (v/v). CM1 corresponds to 50 μM of ASA, and CM5 to 2 μM of ASA.

CM group	ASA pretreatment concentrations (μM)	Final CM applied to PC‐3 (%)
CM1	50	50
CM2	25	50
CM3	10	50
CM4	5	50
CM5	2	50

### 2.4 MTT Test

MTT is a colorimetric assay utilized to assess the metabolic activity of living cells. This assay is based on the capacity of viable cells to reduce 3‐[4,5‐dimethylthiazol‐2‐yl]‐2,5‐diphenyl tetrazolium bromide to formazan, which produces a measurable color change. In this study, PC‐3 prostate cancer cells were seeded at 5 × 10^3^ cells in 100 μL medium per well in two separate 96‐well culture plates (one for 24‐h incubations and one for 48‐h incubations). After the cells were incubated overnight, the medium in each well was aspirated, and the wells were washed once with 100 μL of 1 × PBS.

To evaluate the direct effect of ASA on cell viability, RWPE‐1 and PC‐3 cells were treated with 50, 25, 10, 5, and 2 μM ASA for 48 h under standard culture conditions. After that, the indirect effect of ASA on prostate cancer cell viability was determined using RWPE‐1‐CM. For this purpose, in the 1st group, CM from unpretreated RWPE‐1 cells was added to PC‐3 cells at various ratios (total volume of 100 μL) (CM0). Other groups are divided into 5: CM collected from RWPE‐1 cells pretreated with different concentrations of ASA for 24 h was similarly applied to cancer cells with a total volume of 100 μL (CM1‐5). FBS was added to all treatment groups at 2%. PC‐3 cells cultured in medium alone (without CM) and the DMSO group used for ASA solubility served as the negative control groups. Only blind wells containing medium were included in the blind group. The cells were maintained in the incubator for 24 and 48 h in separately prepared plates. At the end of each incubation period, 10 μL of MTT solution was added to each well, and the plates were incubated at 37°C for 4 h. Isopropyl alcohol was added to the cells to dissolve the formazan crystals, and absorbance was measured at 570 nm using the ELISA reader. Based on the absorbance data, cell viability percentages were calculated according to the relevant formula:

%Viability=[100×(MeanofAbsorbanceoftheChemicallyAppliedGroup−MeanofBlindAbsorbance)/(MeanofAbsorbanceoftheControlGroup−MeanofBlindAbsorbance)].

The data obtained were analyzed with “GraphPad Prism 5” to create dose‐response curves and compare the effects of CM groups on cell viability. In addition, efficacy was evaluated by calculating IC_50_ values (the concentration of CM required to inhibit 50% of cell viability).

### 2.5 Wound Closure Test

Wound closure testing is a standard in vitro technique used to investigate cell movement in a two‐dimensional environment. In this test, mechanical damage creates a space between the cells, which are spread as a single layer on the well surface. Since there is only one space for the cells to move to, they begin moving towards it. In this way, comparative analyses can be made on the migration capabilities of cells in two dimensions [18].

The Wound Healing test was applied to evaluate cell migration capacity. PC‐3 prostate cancer cells were seeded at 5 × 10^4^ cells per well in 24‐well culture plates. The cells were then incubated for 24 h at 37°C in a 5% CO_2_ incubator to allow for the formation of a monolayer. After confirming that the well surface was uniformly covered with cells under the microscope, the wound‐healing process was initiated. A single line was drawn across each well using a sterile 200 μL volume pipette tip, thus creating an artificial “wound”. This gap served as a reference area for evaluating cell migration. After the wound was made, the medium containing the cells was carefully aspirated, and cell debris was removed by washing each well once with 1x PBS. The cells were then divided into two treatment groups. The control group, from which CM was collected from untreated RWPE‐1s at different concentrations, was administered to the cancer cells. In the control and treatment groups, CM was collected from RWPE‐1s pre‐treated with ASA at various concentrations and administered to cancer cells at different concentrations. Three regions were marked for each well, and wound closure status was observed by taking microscope images from the same areas at 0, 24, and 48 h.

### 2.6 Colony Formation Assay

This study employed a colony formation assay to evaluate the long‐term proliferative potential of PC‐3 prostate cancer cells. The cells were seeded at a density of 500 cells per well into 6‐well plates. After seeding, the plates were incubated at 37°C and 5% CO_2_ for 14 days. During this period, the medium was changed every 3 days. The treatment groups were formed: the untreated control group; the CM groups, which contained CM from untreated RWPE‐1 cells; and the ASA (50 µM) group. A mixture of 50% CM and 50% standard culture medium (DMEM containing 10% fetal bovine serum [FBS]) and 1% penicillin‐streptomycin (P/S) was added to the wells designated for the CM groups, resulting in a total volume of 5 mL per well. At the end of the incubation period, each colony was evaluated under a microscope, and acceptance was based on the presence of 50 or more cells. Structures meeting this criterion were classified as colonies. Scratch areas were quantified using ImageJ software. Wound closure was calculated as a percentage of the initial scratch area using the formula:

%WoundClosure=(InitialArea−RemainingArea)×100.

### 2.7 Crystal Violet Staining

Crystal violet dye binds to DNA and proteins, making cells visible purple. In this way, it is possible to clearly visualize and count living cell colonies. At the end of the colony formation test, crystal violet staining was applied to fix the cells and make them visible. First, the culture medium in each well was aspirated; then, the cells were washed once with 1 × PBS. After washing, 500 μL of paraformaldehyde (PFA) solution was added to each well, and the cells were fixed at room temperature for 15 min. Following fixation, the wells were washed twice with 1 × PBS, and 500 μL of crystal violet staining solution was added to each well. After incubating the cells with the dye at room temperature for 10 min, the dye was carefully aspirated. Then, the cells were washed three times with 1 × PBS to remove excess dye. The plates were left to dry at room temperature.

### 2.8 Statistical Analysis

Cell viability data obtained from MTT assays were analyzed using GraphPad Prism 5. Since normality assumptions were not met for all datasets, the non‐parametric Kruskal–Wallis test was applied to compare multiple groups, followed by Dunn's post hoc test for pairwise comparisons against the control group. Data are presented as median ± SEM, with *n* = 3 independent experiments per group. Exact *p*‐values are reported in Section 3 and indicated in the figures, and error bars in all graphs represent SEM to reflect experimental variability.

## 3 Results

**Figure 1:**
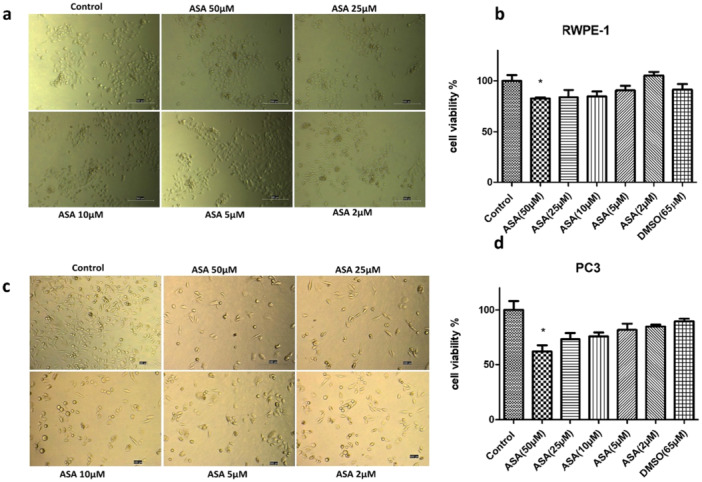
Various concentrations of ASA were administered, and cell viability (expressed as a percentage) was assessed using the MTT assay after a 48‐h incubation. (a) Representative inverted microscopy images of RWPE‐1 cells treated with different ASA concentrations. (b) Quantitative analysis of RWPE‐1 cell viability after 48 h of ASA treatment. (c) Representative inverted microscopy images of PC‐3 cells treated with different ASA concentrations. (d) Quantitative analysis of PC‐3 cell viability after 48 h of ASA treatment. ASA concentrations applied to RWPE‐1 and PC‐3 cells were statistically compared with the respective control groups (*n* = 3, *p < 0.05).

This study aimed to investigate the potential effects of ASA use on prostate cancer prevention. Based on this, the determined ASA concentrations were applied to RWPE‐1, non‐cancerous prostate epithelial cells, and cell viability was assessed using the MTT assay after 48 h. Images of these cells were taken under a microscope before the MTT test. Morphology similar to the control group was observed in all ASA‐treated groups (Figure 1a). Accordingly, cell viability decreased to 82% even at the highest ASA concentration of 50 μM (Figure 1b). For a substance to be considered “cytotoxic,” it must kill more than 30% of cells compared to the control [19]. Each ASA concentration was tested for its effect on PC‐3 cell viability after 48 h to determine the direct impact of ASA on PC‐3 cells. The ASA implemented group morphology was changed, with rounded and cell colonies decreasing (Figure 1c). PC‐3 cell viability was 61.88%, 73.03%, 75.58%, 81.34%, and 84.47% at 50, 25, 10, 5, and 2 μM ASA, respectively (Figure 1d).

**Figure 2:**
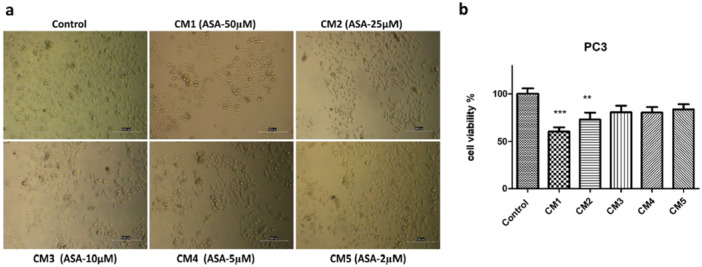
An MTT test was used to determine cell viability (%) at 48 h. (a) 50% of CM‐treated PC‐3 cells are observed. (b) CM1‐5 significantly inhibited cell growth in the prostate cancer cell line PC‐3 in a dose‐dependent manner. Data were collected from three independent experiments for each condition and statistically analyzed by comparing all conditioned medium (CM) experimental groups with the control group (*n* = 3, ***p < 0.001, **p < 0.005).

Consequently, CM were prepared for all determined concentrations. CMs collected from the ASA‐treated groups were applied to PC‐3 prostate cancer cells. After 48 h, an MTT assay was performed to assess cell viability. A decrease in colony‐forming units was observed across all CM‐treated groups, particularly in CM1 and CM2, accompanied by an increase in rounded cell morphology (Figure 2a). Specifically, the cell viability was 60.77% in the CM1 group, 73.37% in the CM2 group, 80.48% in the CM3 group, 81.18% in the CM4 group, and 83.6% in the CM5 group (Figure 2b).

**Figure 3:**
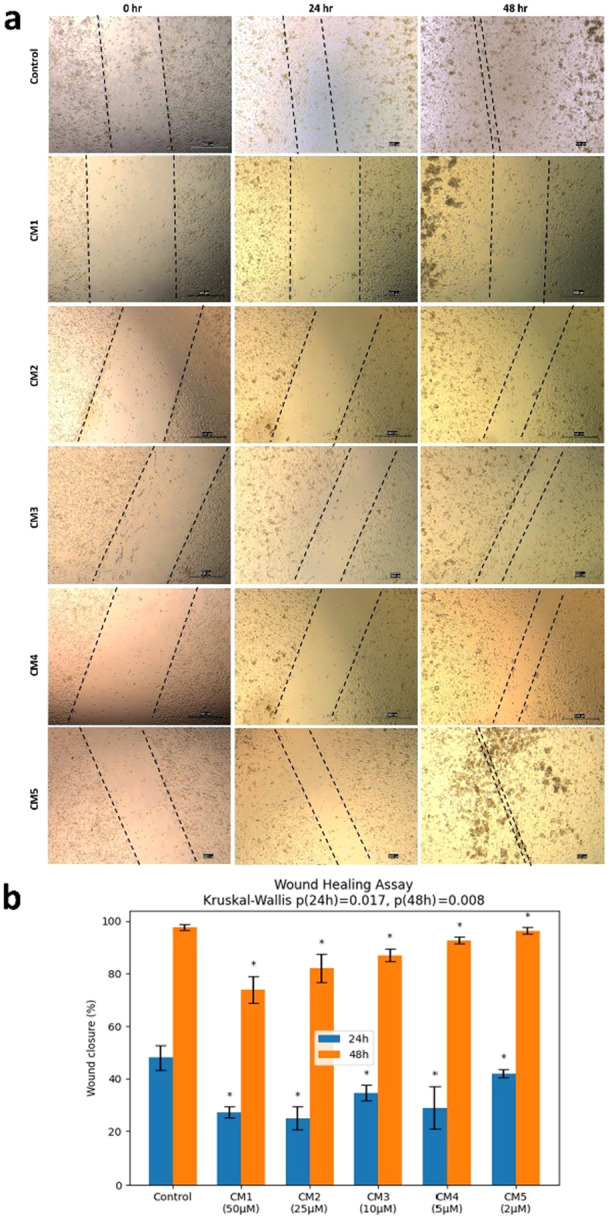
Effect of conditioned media from ASA‐treated RWPE‐1 prostate epithelial cells on PC‐3 cell migration. (a) Representative images of wound‐healing assays showing PC‐3 cell migration at 0, 24, and 48 h following treatment with conditioned media derived from RWPE‐1 cells exposed to different concentrations of acetylsalicylic acid (ASA) (50, 25, 10, 5, and 2 µM). The dashed lines indicate the wound margins. (b) Quantitative analysis of wound closure percentages was determined using ImageJ software. Data represent the percentage of wound closure at each time point relative to the initial wound area (0 h). Conditioned media obtained from ASA‐treated epithelial cells altered the migration dynamics of PC‐3 cells. Statistical analysis was performed using the Kruskal–Wallis test due to the non‐normal distribution of the data. Significant differences compared to the control group are indicated (*p < 0.05).

The migratory capacity of PC‐3 prostate cancer cells following treatment with CM derived from ASA‐treated RWPE‐1 prostate epithelial cells was evaluated using a wound‐healing assay. Representative images of wound closure at 0, 24, and 48 h are shown in Figure 3. Quantitative analysis of wound closure was performed using ImageJ software. At 24 h, the control group showed 43% wound closure, whereas PC‐3 cells treated with CM from ASA‐treated epithelial cells exhibited variable migration rates. The wound closure percentages were 27%, 19%, 31%, 19%, and 42% for CM1 (50 µM), CM2 (25 µM), CM3 (10 µM), CM4 (5 µM), and CM5 (2 µM), respectively. After 48 h, wound closure increased markedly in all groups. The control group reached 96% closure, while CM groups exhibited closure rates of 79%, 88%, 84%, 93%, and 96%, respectively.

**Figure 4:**
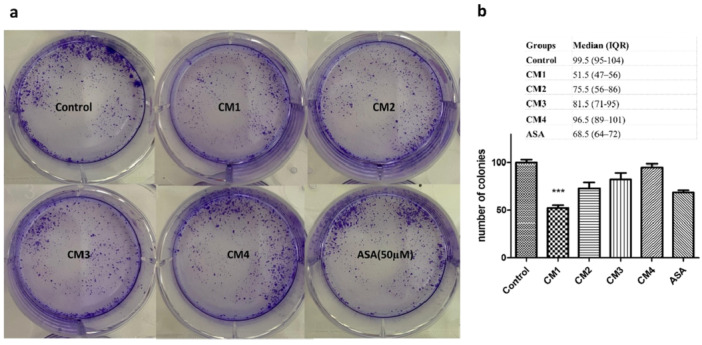
Colony formation assay of PC‐3 prostate cancer cells treated with conditioned media derived from ASA‐pretreated RWPE‐1 cells. (a) Representative images of colony formation in PC‐3 cells. (b) Quantitative analysis of colony numbers. Data are presented as median (IQR) from independent experiments. Statistical analysis was performed using the Kruskal–Wallis test due to non‐normal data distribution. ***p < 0.001 versus control.

The colony formation assay showed that CM derived from RWPE‐1 cells pretreated with 50 μM ASA (CM1) significantly reduced the absolute number of PC‐3 colonies compared with the control group (*p* < 0.05). In contrast, direct treatment with 50 μM ASA did not yield a statistically significant reduction compared with the control group (*p* > 0.05). Importantly, colony numbers in the CM1 group were lower than those in the direct ASA (50 μM) group (*p* > 0.05), indicating that the inhibitory effect mediated by CM was stronger than direct ASA exposure. Quantitative data were expressed as the median (interquartile range) due to non‐normal distribution and analyzed using the Kruskal–Wallis test (Figure 4).

## 4 Discussion

Prostate cancer remains one of the most commonly diagnosed malignancies among men worldwide and continues to represent a significant cause of cancer‐related mortality [20]. Disease progression is influenced not only by tumor cell–intrinsic genetic and epigenetic alterations but also by complex interactions within the tumor microenvironment. Increasing attention has therefore been directed toward therapeutic strategies that modulate tumor–stromal and epithelial–tumor communication rather than exclusively targeting cancer cells in isolation.

Aspirin, a widely used non‐steroidal anti‐inflammatory drug (NSAID), has been associated with reduced cancer incidence and mortality in several epidemiological studies [8, 14]. Its anticancer properties have largely been attributed to cyclooxygenase (COX) inhibition—particularly COX‐2—which reduces prostaglandin synthesis and modulates inflammatory signaling pathways [15]. However, most experimental investigations have focused on the direct cytotoxic or cytostatic effects of aspirin on tumor cells. In contrast, its potential influence on the secretory activity of non‐cancerous epithelial cells has remained insufficiently explored.

In the present study, we investigated whether aspirin pretreatment of RWPE‐1 prostate epithelial cells alters their CM in a manner that can affect PC‐3 prostate cancer cells. Importantly, the ASA concentrations used in RWPE‐1 cells were non‐cytotoxic, maintaining cell viability above 80%, thereby allowing evaluation of secretome‐related effects without inducing overt epithelial damage. Our findings demonstrate that CM derived from ASA‐pretreated RWPE‐1 cells reduced PC‐3 cell viability and migration compared to control CM. Although not all comparisons reached statistical significance across assays, a consistent trend toward decreased proliferative and migratory capacity was observed in the CM1 and CM2 groups. Taken together, the findings of this study suggest that aspirin may influence prostate cancer cell behavior through multiple complementary mechanisms. On one hand, direct exposure of PC‐3 cells to ASA reduced cell viability and clonogenic survival, indicating a direct antiproliferative effect on tumor cells. On the other hand, CM derived from ASA‐treated RWPE‐1 epithelial cells altered the migratory and proliferative responses of PC‐3 cells, suggesting that aspirin may also exert indirect effects by modifying epithelial cell secretory activity. These observations support the concept that aspirin may act not only as a direct inhibitor of tumor cell growth but also as a modulator of epithelial–tumor paracrine interactions within the tumor microenvironment. Previous studies have shown that aspirin can regulate inflammatory signaling pathways, including prostaglandin synthesis and NF‐κB activity, which are known to influence cytokine and growth‐regulatory factor secretion from epithelial cells [15, 21]. Therefore, the combined direct and microenvironment‐mediated effects observed in this study provide a broader perspective on the potential role of aspirin in modulating prostate cancer progression.

The wound healing assay further supported reduced migratory capacity in PC‐3 cells exposed to CM from ASA‐pretreated epithelial cells. Aspirin has previously been reported to interfere with pathways involved in cell motility and extracellular matrix remodeling [14, 22]. While our study did not assess specific molecular mediators, the observed functional effects are consistent with the hypothesis that aspirin‐induced alterations in epithelial‐derived soluble factors may contribute to modified tumor cell dynamics.

Although we did not directly analyze the molecular composition of the CM, several biologically plausible mechanisms may explain how ASA modifies the secretome of non‐cancerous epithelial cells. Aspirin irreversibly inhibits cyclooxygenase enzymes (COX‐1 and COX‐2), leading to reduced prostaglandin synthesis [23]. Prostaglandins are key regulators of inflammatory signaling cascades and can influence cytokine production, growth factor release, and paracrine communication within epithelial tissues. By attenuating prostaglandin‐mediated signaling, ASA may suppress NF‐κB–dependent transcriptional activity, a central pathway regulating inflammatory and secretory genes [24]. Reduced NF‐κB activation may consequently alter the secretion profile of inflammatory mediators, chemokines, and growth‐regulatory factors. Therefore, the functional changes observed in PC‐3 cells exposed to ASA‐modified CM may reflect aspirin‐induced modulation of inflammatory and paracrine signaling pathways in RWPE‐1 epithelial cells. Nevertheless, direct molecular validation through proteomic or cytokine profiling approaches will be necessary to confirm this hypothesis. It is important to emphasize that our study does not characterize the precise molecular components of the altered secretome. Thus, conclusions regarding specific cytokines, growth factors, or downstream signaling pathways cannot yet be drawn. Instead, our findings provide functional evidence that pre‐exposed epithelial cells can influence prostate cancer cell behavior through CM. This microenvironment‐oriented experimental design offers a complementary perspective to traditional monoculture models that focus exclusively on direct drug–tumor interactions.

Clinically, long‐term aspirin use has been associated with modest reductions in cancer mortality and metastatic progression in certain contexts [8, 25]. Although causality and optimal patient selection remain under investigation, our preclinical observations are consistent with the concept that aspirin may exert context‐dependent effects within the tumor microenvironment. The possibility that aspirin modulates epithelial–tumor communication represents an additional layer of complexity beyond its direct tumor‐cell–targeting properties. Future studies should aim to characterize the molecular composition of the aspirin‐modified epithelial secretome using proteomic, cytokine‐array, and signaling‐pathway analyses. Validation of candidate mediators at both protein and transcriptional levels will be essential to elucidate the underlying mechanisms. Expanding this model to additional prostate cancer cell lines may further clarify the generalizability and biological significance of the observed effects.

In conclusion, our findings indicate that aspirin pretreatment of non‐cancerous prostate epithelial cells can influence prostate cancer cell viability and migration through CM. While the mechanistic pathways remain to be fully elucidated, this study highlights the potential importance of epithelial–tumor paracrine interactions in mediating indirect drug effects. It provides a foundation for further investigation of microenvironment‐based strategies in prostate cancer research.

## Author Contributions

Study conception and design: Dilşad Özerkan. In vitro analysis: Selda Durukan and Mahsa Koucheki. Material preparation, experimental work, data collection: Selda Durukan, Mahsa Koucheki. Interpretation of data: Dilşad Özerkan. First draft: Aysel Nesil and Selda Durukan. All authors contributed to the manuscript review and approved the final manuscript.

## AI Use Disclosure

The authors used Grammarly (v1.2.272.1911) in 2026 for language translation and substantial editing to improve the clarity, grammar, spelling, punctuation, and overall readability of the manuscript. Grammarly was used in the preparation and revision of the manuscript, including the Abstract, Introduction, Materials and Methods, Results, Discussion, and Conclusion sections. The authors directed the use of the software, critically reviewed all suggested revisions, and take full responsibility for the final content of the manuscript. The AI‐assisted technology generated no scientific interpretations, data analyses, conclusions, or research findings.

## Conflicts of Interest

The authors declare no conflicts of interest.

## Data Availability

The data that support the findings of this study are available from the corresponding author upon reasonable request.

## References

[1] Z. Khazaei , M. Sohrabivafa , V. Momenabadi , L. Moayed , and E. Goodarzi , “Global Cancer Statistics 2018: Globocan Estimates of Incidence and Mortality Worldwide Prostate Cancers and Their Relationship With the Human Development Index,” Advances in Human Biology 9 (2019): 245, 10.4103/2321-8568.262891.

[2] R. L. Siegel , T. B. Kratzer , A. N. Giaquinto , H. Sung , and A. Jemal , “Cancer Statistics, 2025,” CA: A Cancer Journal for Clinicians 75 (2025): 10–45, 10.3322/CAAC.21871. 39817679 PMC11745215

[3] F. Zorlu , R. T. Divrik , S. Eser , and K. Yorukoglu , “Prostate Cancer Incidence in Turkey: An Epidemiological Study,” Asian Pacific Journal of Cancer Prevention 15 (2014): 9125–9130, 10.7314/APJCP.2014.15.21.9125. 25422189

[4] J. R. Packer and N. J. Maitland , “The Molecular and Cellular Origin of Human Prostate Cancer,” Biochimica et Biophysica Acta (BBA) ‐ Molecular Cell Research 1863 (2016): 1238–1260, 10.1016/J.BBAMCR.2016.02.016. 26921821

[5] J. Miner and A. Hoffhines , “The Discovery of Aspirin's Antithrombotic Effects,” Texas Heart Institute Journal 34 (2007): 179–186. https://pmc.ncbi.nlm.nih.gov/articles/PMC1894700/. 17622365 PMC1894700

[6] D. A. Drew and A. T. Chan , “Aspirin in the Prevention of Colorectal Neoplasia,” Annual Review of Medicine 72 (2021): 415–430, 10.1146/ANNUREV-MED-060319-120913/CITE/REFWORKS. PMC7880546 33035431

[7] A. Grancher , P. Michel , F. Di Fiore , and D. Sefrioui , “Colorectal Cancer Chemoprevention: Is Aspirin Still in the Game?,” Cancer Biology & Therapy 23 (2022): 446–461, 10.1080/15384047.2022.2104561. 35905195 PMC9341367

[8] P. M. Rothwell , M. Wilson , J. F. Price , J. F. Belch , T. W. Meade , and Z. Mehta , “Effect of Daily Aspirin on Risk of Cancer Metastasis: A Study of Incident Cancers During Randomised Controlled Trials,” Lancet 379 (2012): 1591–1601, 10.1016/S0140-6736(12)60209-8. 22440947

[9] E. S. Schernhammer , J. H. Kang , A. T. Chan , et al., “A Prospective Study of Aspirin Use and the Risk of Pancreatic Cancer in Women,” Journal of the National Cancer Institute 96 (2004): 22–28, 10.1093/JNCI/DJH001. 14709735

[10] S. A. Streicher , H. Yu , L. Lu , M. S. Kidd , and H. A. Risch , “Case‐Control Study of Aspirin Use and Risk of Pancreatic Cancer,” Cancer Epidemiology, Biomarkers & Prevention 23 (2014): 1254–1263, 10.1158/1055-9965.EPI-13-1284/14133/P/CASE-CONTROL-STUDY-OF-ASPIRIN-USE-AND-RISK-OF. PMC4091763 24969230

[11] N. R. Cook , I. M. Lee , J. M. Gaziano , et al., “Low‐Dose Aspirin in the Primary Prevention of Cancer: The Women's Health Study: A Randomized Controlled Trial,” Journal of the American Medical Association 294 (2005): 47–55, 10.1001/JAMA.294.1.47. 15998890

[12] P. M. Rothwell , F. G. R. Fowkes , J. F. Belch , H. Ogawa , C. P. Warlow , and T. W. Meade , “Effect of Daily Aspirin on Long‐Term Risk of Death Due to Cancer: Analysis of Individual Patient Data From Randomised Trials,” Lancet 377 (2011): 31–41, 10.1016/S0140-6736(10)62110-1. 21144578

[13] A. Lam , Z. Hao , K. Yiu , et al., “Long‐Term Use of Low‐Dose Aspirin for Cancer Prevention: A 20‐year Longitudinal Cohort Study of 1,506,525 Hong Kong Residents,” International Journal of Cancer 156 (2025): 2330–2339, 10.1002/IJC.35331. 39825684 PMC12008822

[14] D. I. Brixner , D. D. Stenehjem , and C. Ulrich , “Aspirin and Cancer Risk,” JAMA Oncology 2 (2016): 1370–1371, 10.1001/jamaoncol.2016.2308. 27533476

[15] M. J. Thun , E. J. Jacobs , and C. Patrono , “The Role of Aspirin in Cancer Prevention,” Nature Reviews Clinical Oncology 9 (2012): 259–267, 10.1038/nrclinonc.2011.199. 22473097

[16] M. Pandey , M. Rajput , P. Singh , M. Shukla , B. Zhu , and J. Koshiol , “Aspirin and Cancer Survival: An Analysis of Molecular Mechanisms,” Cancers 16 (2024): 223, 10.3390/cancers16010223. 38201650 PMC10778469

[17] S. Y. Wu , J. W. Chen , H. Y. Liu , et al., “Secretory Autophagy Promotes Rab37‐Mediated Exocytosis of Tissue Inhibitor of Metalloproteinase 1,” Journal of Biomedical Science 29 (2022): 103, 10.1186/s12929-022-00886-z. 36457117 PMC9717497

[18] J. E. N. Jonkman , J. A. Cathcart , F. Xu , et al., “An Introduction to the Wound Healing Assay Using Live‐Cell Microscopy,” Cell Adhesion & Migration 8 (2014): 440–451, 10.4161/CAM.36224. 25482647 PMC5154238

[19] ISO 10993‐5:2009 “Biological Evaluation of Medical Devices — Part 5: Tests for In Vitro Cytotoxicity,” (n.d.). Accessed September 11, 2025. https://www.iso.org/standard/36406.html.

[20] H. Sung , J. Ferlay , R. L. Siegel , et al., “Global Cancer Statistics 2020: GLOBOCAN Estimates of Incidence and Mortality Worldwide for 36 Cancers in 185 Countries,” CA: A Cancer Journal for Clinicians 71 (2021): 209–249, 10.3322/caac.21660. 33538338

[21] M. Dovizio , S. Tacconelli , C. Sostres , E. Ricciotti , and P. Patrignani , “Mechanistic and Pharmacological Issues of Aspirin as an Anticancer Agent,” Pharmaceuticals 5 (2012): 1346–1371, 10.3390/ph5121346. 24281340 PMC3816673

[22] H. Maghsoudi , F. Sheikhnia , P. Sitarek , et al., “The Potential Preventive and Therapeutic Roles of NSAIDs in Prostate Cancer,” Cancers 15 (2023): 5435, 10.3390/cancers15225435. 38001694 PMC10670652

[23] J. R. Vane and R. M. Botting , “The Mechanism of Action of Aspirin,” Thrombosis Research 110 (2003): 255–258, 10.1016/S0049-3848(03)00379-7. 14592543

[24] M. Sun , J. Yu , J. Wan , X. Dou , X. Chen , and F. Ye , “Role of Aspirin in Cancer Prevention,” Cancer Treatment and Research Communications 43 (2025): 100884, 10.1016/J.CTARC.2025.100884. 39923320

[25] Y. Cao , R. Nishihara , K. Wu , et al., “Population‐Wide Impact of Long‐Term Use of Aspirin and the Risk for Cancer,” JAMA Oncology 2 (2016): 762–769, 10.1001/jamaoncol.2015.6396. 26940135 PMC4900902

